# Invasive fungal infections in pediatric patients with central nervous system tumors: novel insights for prophylactic treatments?

**DOI:** 10.3389/fonc.2023.1248082

**Published:** 2023-10-27

**Authors:** Natália Dassi, Andrea Maria Cappellano, Adriana Maria Paixão de Sousa da Silva, Nasjla Saba da Silva, Fabianne Altruda de Moraes Costa Carlesse

**Affiliations:** ^1^ Pediatric Oncology, Pediatric Oncology Institute-GRAACC (IOP-GRAACC)/Federal University of São Paulo, São Paulo, Brazil; ^2^ Pediatric Infectology, IOP-GRAACC/Federal University of São Paulo, São Paulo, Brazil

**Keywords:** invasive fungal infections, pediatric central nervous system tumors, infectious diseases, prophylactic treatments, Intracranial tumors

## Abstract

**Background and aims:**

Invasive fungal disease (IFD) poses significant morbidity and mortality risks, especially in pediatric patients with neoplastic diseases. However, there is a notable lack of data concerning patients with central nervous system (CNS) tumors. Considering vulnerability factors to infections such as neutropenia, corticosteroids, chemotherapy, surgical interventions, and others, this study aims to evaluate the incidence of IFD in pediatric patients with CNS tumors and determine appropriate indications for prophylactic measures. This is a single-center, retrospective study conducted between 2011 and 2022 at the Pediatric Institute of Oncology (IOP-GRAACC-UNIFESP).

**Results:**

A total of 38 cases of IFD were diagnosed in 818 children with CNS malignancies (4,6%). The mean age was 3.5 years (0.4-28y), with 22 (57.9%) male patients. Embryonal tumors (18/38, 47.3%) were the most prevalent CNS tumors, followed by low-grade gliomas (13/38, 34.2%). All episodes met the EORTC IFD criteria, and 36/38 (94.7%) were proven. Invasive yeast infections (33/36, 91.6%), predominantly *Candida* (30/33, 90.9%), were the most common diagnosis. In total, 25 patients (25/38, 65.8%) were receiving chemotherapy, with 13 of them having embryonal tumors. A total of 11 infants were in the Head Start scheme, resulting in a high prevalence of IFD in these group of patients (11/58, 18.9%). In total, 13 (13/38, 34.2%) patients underwent neurosurgery, mostly ventricular-peritoneal shunts revisions (10/13, 76.9%). Nine (9/38, 23.7%) were with prolonged use of corticosteroids, eight of them associated with neurosurgery.

**Conclusion:**

Routine systemic antifungal prophylaxis based solely on diagnosis is not recommended for low-risk cases. Evaluating patient- and treatment-specific risk factors is crucial in infants undergoing high-dose chemotherapy with expected neutropenia and in patients requiring prolonged corticosteroid therapy alongside neurosurgical procedures.

## Introduction

Invasive fungal disease (IFD) poses significant morbidity and mortality risks, especially among pediatric patients with neoplastic diseases (1). Furthermore, it is well established that patients with hematologic malignancies and those undergoing allogeneic bone marrow transplantation have a high risk of developing infection and an indication of primary prophylaxis (1, 2). Few studies describe the incidence of these diseases in patients with solid tumors (3, 4) and there is an absence of information regarding pediatric patients with central nervous system (CNS) tumors. Considering other vulnerability factors to infections such as prolonged neutropenia, central venous catheter use, corticosteroid administration, cytotoxic chemotherapy, surgical interventions, and individual comorbidities (2, 5), this study aims to assess the incidence of IFD in pediatric CNS tumor patients. Furthermore, the study seeks to elucidate clinical characteristics, predisposing factors, diagnostic approaches, treatment modalities, outcomes, and indications for prophylaxis within this specific patient population.

## Methods

This retrospective, single-center, observational study was conducted on children diagnosed with CNS malignancies and IFD between 2011 and 2022. The study was carried out at the Pediatric Oncology Institute (IOP-GRAACC), affiliated with the Federal University of São Paulo. The Institute operates as a tertiary university hospital and handles approximately 100 new neuro-oncology cases per year. These cases are covered by the Brazilian Unified Health System (SUS), which offers universal access to healthcare for all citizens. Additionally, some patients have private health insurance that covers their treatment costs.

Patient characteristics studied included demographic information, CNS tumor details, treatment modality (such as surgery, chemotherapy, and/or radiotherapy, but excluding the duration of bone marrow transplant), predisposing or risk factors (such as corticosteroid use, prolonged neutropenia, extended hospitalization, presence of central venous catheters, and prior antibiotic usage), IFD diagnosis, antifungal treatment, and clinical outcomes.

The systematic review by Fisher et al. showed that the definitions for some risk factors are not well established. In our cohort, prolonged neutropenia was characterized by an absolute neutrophil count of less than 500 μL for more than 10 days. Lymphopenia was defined as a lymphocyte count of less than 1000 cells/mL. Prolonged hospitalization was categorized as a hospital stay of more than seven days, and prolonged use of corticosteroids was identified as a daily dose of dexamethasone exceeding 0.6mg/m2 for more than 21 days.

Episodes of IFD were categorized as possible, probable, or proven based on the international consensus criteria of the European Organization for Research and Treatment of Cancer (EORTC) (6, 7).

All data were systematically tabulated, and descriptive analyses were employed to report the demographic, clinical, and mycological characteristics of the entire study population. Ethical approval for research involving human subjects was obtained from the study center before the start of the study itself.

## Results

### Patient characteristics

During the 11-year observation period, 38 cases of IFD were identified among a total of 818 children with CNS malignancies, representing a prevalence of 4.6%. The mean age was 3.5 years, ranging from 0.4 to 28 years, and the majority of cases, 22, were from male subjects (57.9%). The most frequently encountered CNS tumors were embryonal, accounting for 18 out of 38 cases (47.4%), with medulloblastoma being the predominant subtype (11/18, 61.1%). Low-grade gliomas (LGG) followed closely, accounting for 13 out of 38 cases (34.2%).

Of the total cases, 94.7% (36 out of 38) met the EORTC criteria (7) for proven IFD, while 5.3% (two out of 38) were categorized as probable, and none met the criteria for possible IFD. Within the subset of proven IFD episodes, the most prevalent diagnosis was an invasive yeast infection, accounting for 33 out of 36 cases (91.6%), with *Candida* infections being the predominant subtype in this category (30 out of 33, 90.9%). Among *Candida* infections, *C. albicans* and *C. parapsilosis* were the most common species, representing 40% (12 out of 30) of cases each. Three out of 36 (8.3%) proven episodes were attributed to molds, specifically *Fusarium oxysporum*. Both probable IFD episodes were identified as suspected invasive pulmonary aspergillosis, supported by positive galactomannan results and characteristic imaging findings.

The sites of infection were distributed as follows: bloodstream infections accounted for the majority, with 32 cases (84.2%), central nervous system (CNS) infections were observed in five cases (13.1%), and pulmonary infections were identified in two cases (5.3%). Additionally, one patient presented with both CNS and bloodstream infections simultaneously.

Cohort characteristics are shown in **Table 1**.

**Table 1:** Underlying disease and epidemiology of IFD in 38 patients from our cohort.

Central nervous system tumor diagnosis Embryonal Tumors Gliomas Low-grade glioma High-grade glioma Ependymoma Plexus Choroid carcinoma Germ cell tumor	18 (47.4%) 14 (36.8%) 13/14 (92.8%) 1/14 (7.2%) 3 (7.9%) 2 (5.3%) 1 (2.6%)
Fungal species Yeasts *C. albicans* *C. parapsilosis* *C. tropicalis* *C. lusitanae* *C. pelliculosa* *Trichosporon japonicum* *Cryptococcus neoformans* *Exophialia spp* Molds *Fusarium oxysporum* Probable Aspergillus	12 (31.6%) 12 (31.6%) 3 (7.9%) 2 (5.3%) 1 (2.6%) 1 (2.6%) 1 (2.6%) 1 (2.6%) 3 (7.9%) 2 (5.3%)
Site of IFD* Bloodstream Central nervous system Pulmonary	32 (84.2%) 5 (13.1%) 2 (5.3%)

*One patient had both bloodstream and CNS infection.

### Risk factors

Of the study participants, 25 out of 38 (65.8%) were receiving chemotherapy. Of these, 13 had embryonal tumors, and 11 were infants enrolled in the *Head Start* backbone scheme (8). Regarding gliomas, 13 out of 14 low-grade gliomas (LGG) were identified, with six of them receiving chemotherapy as follows: three were following the *Roger Packer* protocol (9) as their first-line treatment, one was on vinorelbine (10), and another on temozolomide (11), both irresectable/refractory cases. One case of infant-type hemispheric glioma was treated with the *Baby-POG protocol* (12). Furthermore, neurosurgical interventions had been performed in 13 out of 38 cases (34.2%). Most of these procedures involved ventricular peritoneal shunt revision, accounting for 10 out of 13 cases (76.9%). The other three cases were tumor resections. One patient (one out of 38, 2.6%) with posterior fossa ependymoma received focal radiotherapy, while five patients (five out of 38, 13.1%) were out of treatment/in the follow-up phase.

Among the additional risk factors observed, all patients had central venous catheters. Nine out of 38 patients (23.7%) were identified as having a prolonged course of corticosteroids of more than 21 days. A total of 30 patients (78.9%) were receiving broad-spectrum antibiotics, with eight of them treated for bacterial bloodstream infections. In total, 22 patients (57.9%) had a prolonged hospitalization of more than 7 days, with the majority of these cases exceeding 30 days. Of these patients, 11 were admitted to the intensive care unit (ICU). A total of 10 patients (26.3%) had an absolute neutrophil count of less than 500 cells/mL, while 22 patients (57.9%) had a lymphocyte count of less than 1000 cells/mL. Additionally, four patients (10.5%) were treated for typhlitis, but none had undergone abdominal surgery.

**Figure 1:**
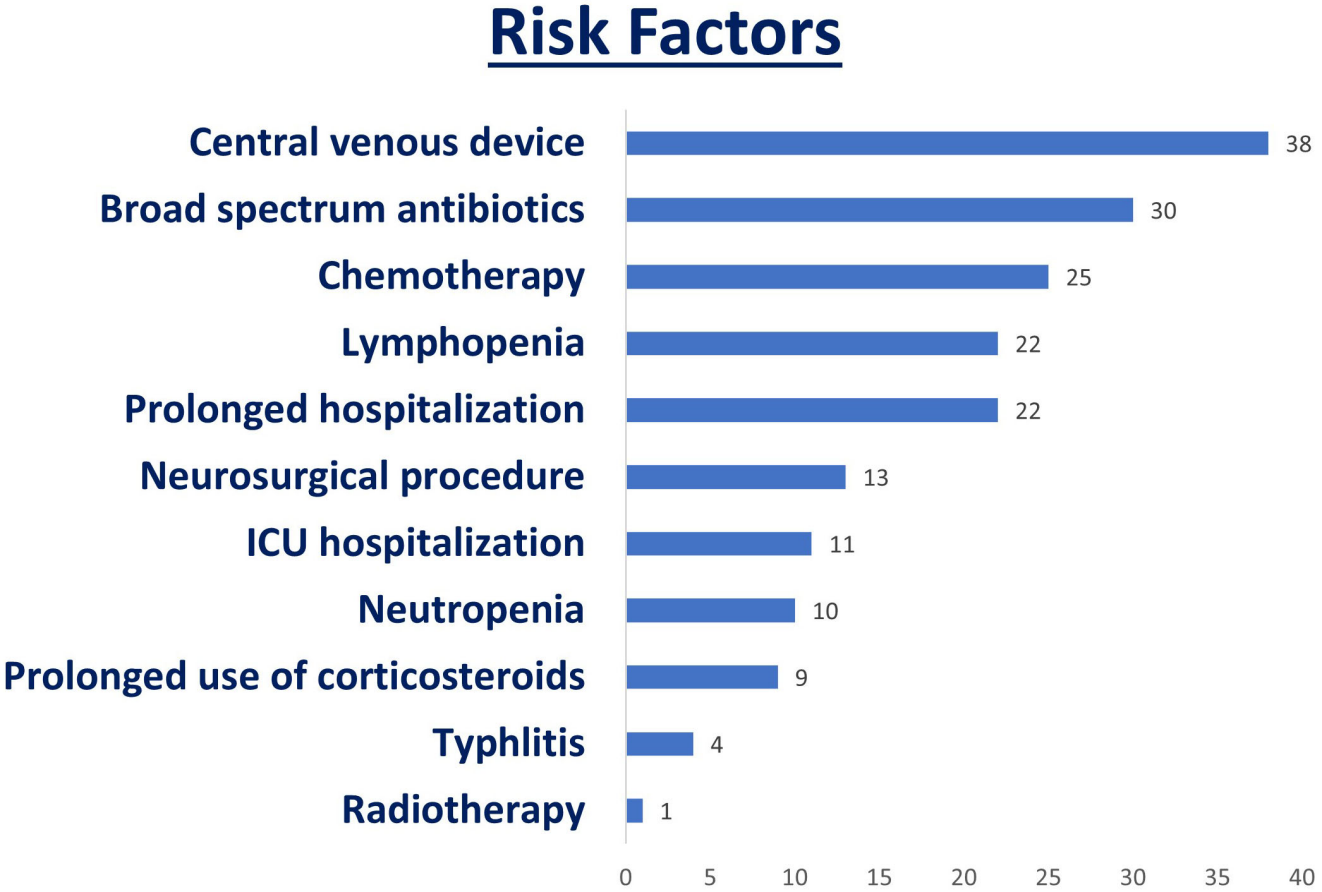
Risk factors associated with IFD.

Risk factors associated with IFD are shown in (**Figure 1**), and their associations are shown in **Table 2**.

**Table 2:** Correlation between the number of associated risk factors and patients with IFD.

Number of risk factors associated	Number of patients n= 38 (100%)
One risk factor Two Three Four or more	3 (7.9%) 4 (10.5%) 12 (31.5%) 19 (50%)

### Treatment and outcomes

In our cohort, the primary drugs used in the treatment of IFD were amphotericin, azoles, and echinocandins, as detailed in **Tables 3** and **4**.

**Table 3:** Antifungal treatment for yeasts.

Antifungal Treatment (Yeast)	Number of patients n=33 (100%)
Amphotericin	14/33 (42.4%)
Echinocandin	12/33 (36.4%)
Azoles Fluconazole Voriconazole	4/33 (12.1%) 3/4 (75%) 1/4 (25%)
Combined antifungal therapy	3/33 (9.1%)

**Table 4:** Antifungal treatment for molds.

Antifungal Treatment (Mold)	Number of patients n=5 (100%)
Amphotericin	2/5 (40%)
Azoles Voriconazole	3/5 (60%) 3/3 (100%)

Among the cases involving yeasts, seven out of 33 (21.2%) patients died; however, only two of these deaths were attributed to invasive fungal disease (IFD). One patient with medulloblastoma developed a bloodstream infection with *C. tropicalis*, and another patient with atypical teratoid/rhabdoid tumor (ATRT) developed a bloodstream infection with *C. albicans* and *Klebsiella pneumoniae*, leading to septic shock and typhlitis. Another patient with relapsed leptomeningeal medulloblastoma passed away due to progressive disease and *Cryptococcus neoformans* IFD affecting the CNS and bloodstream. The remaining four patients died in a palliative care context, unrelated to IFD. Regarding molds, two out of five cases (40%) were affected by *Fusarium oxysporum* IFD but ultimately died due to progressive disease. Therefore, within the entire cohort, nine patients (nine out of 38, representing 23.7%) succumbed to various causes, with only two of these deaths attributed to IFD. The other seven patients passed away in a palliative care setting due to progressive disease.

## Discussion

This study provides insight into the epidemiology and treatment of invasive fungal disease (IFD) in pediatric patients with CNS malignancies. Our findings reveal a notable incidence of proven instances of IFD in this patient group, which has traditionally been categorized as low-risk based on their diagnosis alone (13).

The true prevalence of invasive fungal disease (IFD) in children with solid tumors remains underexplored, as highlighted by recent studies by Ruijters et al. (3, 4). Additionally, discussions of IFD cases in the context of CNS tumors are often limited to scenarios involving autologous stem cell transplantation (ASCT) (14). This study aimed to assess the necessity for antifungal prophylaxis in the context of CNS malignancies, excluding the risk associated with ACST that may occur during CNS tumor therapy. This is the rationale behind the exclusion of this specific period from the study.

In the spectrum of fungal infections observed, *Candida* species or invasive candidiasis/candidemia were prevalent in the majority of cases (30/38, 78.9%), a trend consistent with studies in hematological diseases (5). Notably, non-albicans *Candida* species accounted for a significant proportion of these cases (18/30, 60%), consistent with the global epidemiological shift toward an increased prevalence of non-albicans species (15). Specifically, *C. parapsilosis* was the most frequently isolated non-albicans species overall (12/30, 40%), an association first reported in solid tumors by Bartlett et al. (15). However, this study did not specify the subtype of solid tumor in which this association was observed, and CNS tumors represented a minority of cases in their cohort. Given the affinity of *C. parapsilosis* for central venous access devices (15) and the presence of at least this risk factor in most oncology patients, including in our cohort, it is crucial to consider the direction of prophylactic measures.

Mold pathogens other than *Aspergillus* are on the rise, constituting 10-25% of invasive mold disease in patients with hematological malignancies or post-hematopoietic stem cell transplantation (HSCT) and carrying a high mortality rate (16). *Fusarium* spp. accounted for 60% (3/5) of the invasive molds identified in our study, despite the small number, which differs from the predominance of *Aspergillus* in hematological cases (17). These patients were previously reported by Carlesse et al. in 2013 (18), due to a hospital outbreak of *Fusarium oxysporum* catheter-related fungemia. This is noteworthy as it deviates from the typical route of infection for this fungal pathogen, which is primarily through the respiratory tract (16). Importantly, we have observed complete clinical recovery following the removal of the central venous device and appropriate antifungal therapy (18). Two patients, one with ependymoma and one with choroid plexus carcinoma, were treated with amphotericin but later succumbed to progressive tumor disease.

Exposure to corticosteroids and antibiotics, central venous devices, prolonged hospitalization (including in the ICU), treatment regimens, and blood counts have all been associated with an increased risk of IFD (2). This is particularly relevant in the context of low-risk based on diagnosis alone. However, determining the precise impact of each factor can be challenging due to their overlapping effects, as demonstrated in our study.

Of the 38 patients, 25 (65.8%) developed IFD after undergoing chemotherapy regimens, with a notable incidence among infants receiving the Head Start backbone chemotherapy regimen, seven of whom had both neutropenia and lymphopenia, and six of whom also required prolonged hospitalizations. During the study period, 58 infants were subjected to this protocol, resulting in a high prevalence of IFD in this patient group (11/58, 18.9%). According to the 2020 guidelines (1), an incidence of ≥10% is typically considered high risk for IFD, and primary antifungal prophylaxis is strongly recommended to reduce morbidity and mortality associated with the disease. Therefore, this group of patients may have benefited from primary antifungal prophylaxis for *Candida* species, given the expected neutropenia, prolonged duration, depth, and/or association with fever following intensive chemotherapy, all of which are highly correlated with IFD. Lymphopenia has also been identified as a risk factor for IFD in adult HSCT recipients (2). In this patient group, these risk factors often overlap, although we describe an additional seven cases with lymphopenia alone, three of which had prolonged hospitalizations.

Exposure to corticosteroids has been associated with an increased risk of IFD in patients with hematological malignancies and those undergoing HSCT (2). When considering only neurosurgical procedures, our study revealed a relatively low rate of fungal infections, accounting for less than 1% of cases during the study period. All the described procedures followed infection control standards, such as a sterile neurosurgical environment and antibiotic prophylaxis. However, it is worth noting that within our study cohort, eight out of nine patients who experienced prolonged corticosteroid use were also associated with neurosurgical procedures, with VP shunt revisions being the most common. Three of these patients developed CNS fungal infections, two of which were caused by *Candida albicans*. These factors collectively underscore the significance of this association, as it has been described that *Candida* virulence genes can be upregulated in patients receiving steroids, particularly in cases involving hydrocephalus-related conditions (19). This upregulation may contribute to the mortality rate associated with *Candida* CNS infections, which typically ranges from 10% to 33% (20). In light of these considerations, patients subjected to high doses of corticosteroids due to VP shunt dysfunction are categorized as a high-risk group. For such individuals, we recommend the implementation of primary antifungal prophylaxis for *Candida*, such as fluconazole, during the period of dysfunction and neurosurgery. This prophylactic measure may be particularly important, especially in regions with a high prevalence of VP shunt usage, which is often the case in low- to middle-income countries (21).

The mortality rate associated with IFD in our cohort demonstrated more favorable outcomes compared to many of the previously reported studies, as indicated in the systematic review conducted by Ruijters et al. The mortality rates in these studies varied widely, ranging from 0% to 66.7%. Notably, only one study reported no deaths associated with IFD (4). This difference in mortality rates reflects the substantial improvements in supportive care, diagnostic capabilities, and therapeutic strategies that have been achieved.

Patients undergoing intensive chemotherapy for conditions such as acute myeloid leukemia (AML), high-risk acute lymphoblastic leukemia (ALL), and recurrent acute leukemia are strongly recommended to receive primary antifungal prophylaxis (1, 13). This recommendation is particularly pertinent in cases of AML, where systemic antifungal prophylaxis is advisable for children undergoing treatment that is expected to result in profound and prolonged neutropenia. In the context of ALL, the decision to administer systemic antifungal prophylaxis should be adapted to the specific treatment protocol, taking into account the varying risk of IFD across protocols and treatment phases. Additionally, according to these guidelines, the choice of antifungal agent should consider factors like local epidemiology, potential drug interactions, adverse effects, and cost-effectiveness. Mold-active agents are typically recommended in these cases (1, 13). Conversely, routine prophylaxis is not generally recommended for children with cancer who are at low risk for IFD. Instead, a personalized assessment based on individual risk factors should be conducted (1, 13). According to our cohort profile, for children with CNS tumors, active agents effective against yeasts, particularly *Candida* species, are recommended in two specific scenarios. First, in infants with embryonal tumors undergoing high-dose chemotherapy during the neutropenia phase, and second, when high-dose corticosteroids are used in cases of ventriculoperitoneal (VP) shunt dysfunction. Fluconazole, with its favorable cost-effectiveness, low potential for drug interactions, and manageable side-effect profile, is a suitable choice. However, local epidemiological data should be considered, and prospective analyses should be conducted to inform decision-making.

Given the study design and the substantial heterogeneity among patients with respect to risk factors (such as age, CNS tumor diagnosis, and treatment modalities), it was not possible to perform additional statistical associations. Despite this statistical limitation, we emphasize the importance of describing various risk factors, as they may offer valuable insights for prospective and controlled studies in the future.

In accordance with the guidelines set forth by Lehrnbecher et al., and considering the specific characteristics of our cohort, we have concluded that routine systemic antifungal prophylaxis is not advisable for patients at low risk based solely on their diagnosis. However, it is important to assess individual patients and treatment-related risk factors. In cases involving infants undergoing high-dose chemotherapy with profound and prolonged neutropenia or patients requiring prolonged corticosteroid use alongside neurosurgical procedures, the use of antifungal prophylaxis may provide potential benefits.

## Data availability statement

The raw data supporting the conclusions of this article will be made available by the authors, without undue reservation.

## Ethics statement

The studies involving humans were approved by Ethics and Research Committee of the Federal University of Sao Paulo - CAAE 67891023.0.0000.5505. The studies were conducted in accordance with the local legislation and institutional requirements. The ethics committee/institutional review board waived the requirement of written informed consent for participation from the participants or the participants’ legal guardians/next of kin because Data Use Commitment Term (TCUD) was used.

## Author contributions

ND, AC, and FC: These authors contributed equally to this work and share first authorship. AS and NS: contributed to conception and design of the study. All authors contributed to the article and approved the submitted version.
